# Simplifying and Testing the Psychometric Psychiatric Patients’ Fall Risk Scale: An Analysis of One-Year Admissions

**DOI:** 10.3390/healthcare9091119

**Published:** 2021-08-30

**Authors:** Yu-Hui Shen, Chia-Chi Hsieh, Ming-Tsung Lee, Wen-Chin Lee, Bih-O Lee

**Affiliations:** 1 Chang Bing Show-Chwan Memorial Hospital, Changhua 505, Taiwan; whershen@yahoo.com.tw (Y.-H.S.); chachi27@gmail.com (C.-C.H.); wenchin2010@gmail.com (W.-C.L.); 2 Research Assistant Center, Show Chwan Memorial Hospital, Changhua 500, Taiwan; lee6717kimo@yahoo.com.tw; 3 Department of Nursing, Hungkuang University, Taichung 433, Taiwan; 4 School of Nursing, Kaohsiung Medical University, Kaohsiung 807, Taiwan

**Keywords:** psychiatric fall risk assessment, patient safety, psychometric property test

## Abstract

This study aimed to simplify the number of items evaluated by fall risk assessment scales for psychiatric patients, conduct associated reliability, validity, and receiver operating characteristic analyses, and determine fall predictors for psychiatric patients. This methodological study was conducted in a hospital specializing in psychiatry, using data from 1101 patients who were hospitalized in 2018. This fall risk assessment scale was modified by the hospital for use in psychiatric patients. The mean age of the sample population was 44.88 (SD = 12.05) years, and the mean duration of hospital stay was 44.04 (SD = 48.14) days. Men comprised 66% of the study population, and women were 34%. Item reduction, psychometric testing for validity and reliability, and receiver operating characteristic analyses were conducted. Logistic regressions were used to analyze fall predictors, including “having anti-epileptic drugs”, “need for walking aids”, and “having experienced fall occurrence within one year”. This study successfully reduced the number of items assessed by the previous scale. The optimal cutoff point was reduced, and the sensitivity and accuracy of the newly revised scale were good. Three fall predictors for psychiatric patients were identified. The revised scale can facilitate the rapid and accurate identification of high-risk, fall-prone psychiatric patients by psychiatric nurses. Hospital information screening should include each patient’s fall history.

## 1. Introduction

The number of patients with psychiatric illnesses has increased worldwide. Falls represent a persistent problem in psychiatric inpatient settings [1]. Scholars have estimated that falls experienced by patients with psychiatric illnesses are associated with medical costs as high as USD 574,000 per psychiatric unit each year [2]. An Australian study found that the medical expenses due to falls amounted to approximately USD 3906 per person, with expenses related to falls accounting for 25% of all national healthcare expenditures on average, and women incurring higher costs than men, at USD 4211 compared with USD 3366 per person, respectively [3]. A study from Taiwan showed that more severe fall injuries were associated with higher associated medical expenses, with the per-person amount reaching USD 546–3651 [4]. In addition, fall-prone individuals are most commonly found among older and psychiatric patients [5]. Patients with psychiatric illnesses are at particular risk for repeated falls due to various factors, including unsteady gait due to the use of multiple antipsychotic drugs, confusion, delusions, and cognitive impairment, among other things [6,7]. Therefore, the costs associated with psychiatric patient falls are a driving force for healthcare managers, who hold hospitals responsible for preventable injuries.

According to reports by the Taiwan Clinical Performance Indicator (TCPI), the rate of fall occurrence in hospital psychiatric departments between 2016 and 2018 was 0.11%, which was higher than that in general wards (0.06%) [8]. Psychiatric inpatients experienced falls at a rate of 13–25 people per 1000-person days, with 4 out of 1000 patients requiring surgery as a result. Based on the Taiwan Patient Safety Reporting System, psychiatric hospitals reported a total of 3835 falls, accounting for 37.9% of all falls, which represented the second-highest percentage (behind harmful events) of abnormal events [8]. More recently, a large study analyzing six years of data from a US national database of psychiatric hospital inpatient units found that 21.6% of psychiatric patients experienced injures after falls. Only 7% of fall patients were receiving assistance from nurses at the time of their fall. The high fall rate and high fall injury reported by psychiatric departments remain ongoing concerns [1].

Eight major goals have been established for inpatient safety in Taiwan. Among them, preventing falls and reducing the severity of injuries constituted key issues [8]. According to the International Patient Safety Goals (IPSG), reducing fall risk remains a key issue for international patient safety [9]. To prevent fall occurrence and reduce injury severity among patients, critical issues identified in a review published by the Taiwan Ministry of Health and Welfare included the adoption of fall risk assessment tools with good reliability and validity by all hospitals, conducting early and accurate screening of patients to identify those at high risk of falls, providing appropriate health education to patients, and taking preventive measures to reduce fall occurrence [8].

Psychiatric inpatients represent a high-risk group for falls. For example, Lu et al. [6] found that patients with psychiatric illnesses often suffer from cognitive impairments and dementia, resulting in decreased extremity control and a greater likelihood of falls. Illness-related factors that can affect fall occurrence among psychiatric patients include confusion, insomnia, dizziness, migraines, physical weakness, movement disorders, postural hypotension, hearing and visual impairments, and incontinence or diarrhea. Studies have found that patients who have experienced repeated falls (more than one) within the previous year were 5.05 times more likely to experience another fall compared with patients who have not experienced a recent fall [6,7]. In addition, psychiatric patients over the age of 65 have a higher incidence of falls when using antipsychotic drugs [10]. Another study also found that patients accompanied by companions were 0.34 times less likely to fall than those without companions [4]. Falls among psychiatric patients have thus incurred substantial medical costs [11]. However, research examining the factors that contribute to fall occurrence among psychiatric patients remains relatively lacking.

A survey of fall risk assessment scales used around the world revealed that a majority consisted of assessment tools targeting older individuals, such as the Hendrich II Fall Risk Model [12]. Although other fall assessment tools are currently verified and in use, they are more suitable for use in patients treated by departments of medicine and surgery, such as the St. Thomas Risk Assessment Tool (STRATIFY). By contrast, fall assessment tools dedicated to psychiatric patients are rare [13]. Commonly used assessment tools for predicting which psychiatric patients are at high risk of falling include the Edmonson Psychiatric Fall Risk Assessment Tool (EPFRAT) [14] and the Wilson–Sims Fall Risk Assessment Tool (WSFRAT) [15]. For these two fall risk assessment scales, the numbers of items assessed are 10 and 15, respectively, but the sample sizes used to test them were relatively small, at 138 and 50, respectively, which is not ideal [16]. In addition, a study in Taiwan compared the sensitivity, specificity, and accuracy of the Psychiatric Inpatient Fall Risk Assessment Tool against those of the WSFRAT and found that the sensitivity levels of the scales were less than ideal, with large differences in cutoff scores [17].

Based on a large-scale study, which included 1159 psychiatric units in 720 hospitals, approximately 22% of all psychiatric patients experienced traumatic injuries due to falls. Only 7.0% of these falls were assisted by nursing staff [1]. A review paper summarized that more than 76% of all hospitalized patients were not assessed for risk of falling. Thus, assessing the sensitivity and specificity of fall risk assessment tools is important for clinical settings [18]. However, the amount of research that focused on inpatient safety in psychiatric units has been limited.

Psychiatric falls and associated injuries have aroused special attention. Psychiatric falls are persistent problems that require evidence obtained from larger datasets to develop fall prevention intervention strategies. Before evidence to support effective fall prevention strategies can be obtained among patients with psychiatric illnesses, a simple and precise tool for the early detection of fall risks may be necessary. The aims of this study were to simplify the number of items required for evaluation in fall risk assessment scales for psychiatric patients; conduct associated reliability, validity, and receiver operating characteristic analyses; and determine fall predictors for psychiatric patients.

## 2. Method

### 2.1. Design

This methodological study included the psychometric testing and modification of fall risk assessment scales for psychiatric patients.

### 2.2. Research Setting

The hospital where this study was conducted is a teaching hospital in Taiwan that specializes in psychiatry. The hospital houses a total of 1000 beds, including 400 in the psychiatry department, consisting of 100 beds for acute patients and 300 beds for patients who require long-term care.

### 2.3. Modification of the Scale

**Figure 1:**
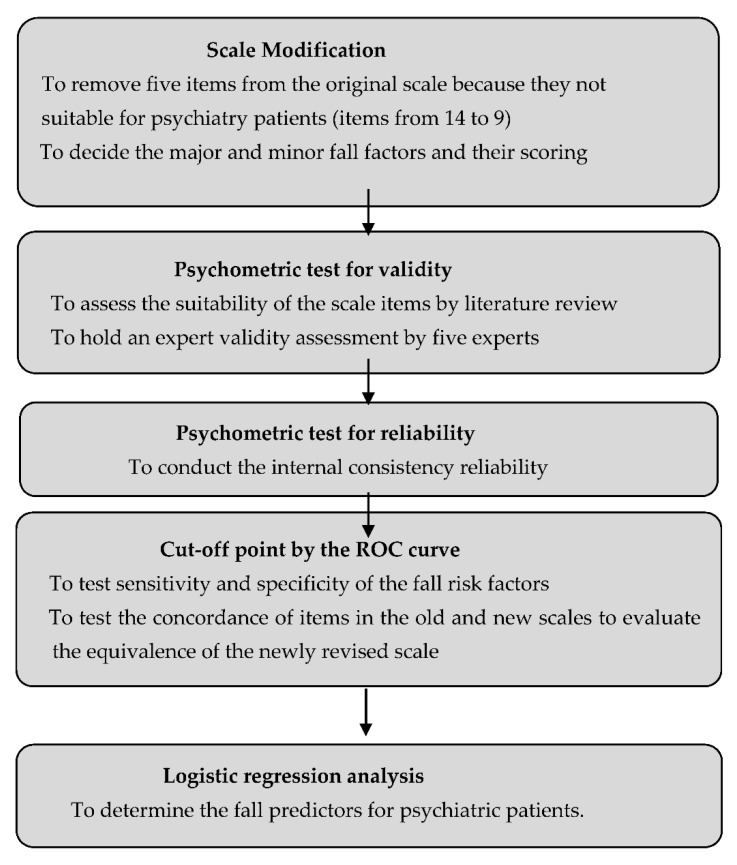
The different phases of the study.

This fall risk assessment scale was specifically modified by the hospital for use in psychiatric patients. In line with national policies, the study hospital used the Morse scale, the STRATIFY scale, the Hendrich II scale, and existing literature as the foundation for establishing its previous fall risk assessment scale for all patient groups in 2008. To effectively prevent falls, the government advocated the need for every hospital to develop realizable fall risk assessments. The original scale, which has not been tested for reliability and validity, has been used for several years. The psychometric testing and modification of the scale were, therefore, necessary to remain in compliance with the government’s policy. Figure 1 shows the different phases of this study.

To facilitate the ability for a psychiatric nurse to quickly assess a patient’s fall risk, the nursing managers opted to remove items from the original scale rather than add items. The original scale consisted of 14 items, including major and minor fall factors. At the time, based on expert opinions, the factors “older than 65 years”, “unsteady gait or lower limb muscle strength ≤ 3 points”, and “consuming drugs affecting conscious activity” were considered the most important factors affecting fall risk. These items were worth 4 points each, whereas the remaining items were worth 1 point each. The highest possible score for the scale was 23 points, with the lowest possible score equal to 0 points. A score of 8 points or above was considered indicative of a high risk of falling. At the time, the cutoff score was determined via consensus; however, the reliability and validity of the scale were not tested.

The fall risk assessment scale is a general assessment tool that was extensively used by the study hospital. When used for psychiatric patients, the assessment items did not match clinical attributes, as some items were not applicable, such as leaving the bed for the first time following surgery or childbirth and abnormal sensations in the foot. The nurse–patient ratio between psychiatric nurses and chronic psychiatric patients was 1:60. Therefore, the assessment items required simplification and revision to more accurately identify psychiatric patients at high risk of falling.

### 2.4. Psychometric Testing

The steps used during the revision process for determining the scale assessment items and conducting psychometric testing were (a) assessing the suitability of scale assessment items through a literature review; (b) conducting an expert validity assessment involving five experts, including a deputy director of medical treatment quality at the research hospital, a psychiatrist, a professor working as an academic consultant in the nursing department, a psychiatric care supervisor, and a nursing quality manager; and (c) testing the internal consistency reliability, fall risk score cutoff point, and factors associated with fall prediction after removing incongruent assessment items.

### 2.5. Measures

Data collected using the original 14-item fall risk assessment scale were assessed. The items removed from the original scale were vision (total blindness, partial blindness, or complaints of blurred vision), difficulty moving or standing up, depression, abnormal sensations in the feet, and leaving the bed for the first time following surgery or childbirth. The nine items that comprised the modified assessment scale are listed in the result section.

### 2.6. Data Collection

The goal of modifying the scale was established in 2018; therefore, the nurse managers opted to utilize data associated with psychiatric inpatients starting in 2018, as the hospital’s fall data were updated that year. With the permission of the nursing department of the study hospital and the information management committee, medical data were retrieved from the medical treatment electronic data system, and data regarding psychiatric inpatients who were hospitalized between 1 January and 31 December 2018 were collected. A total of 1101 patient cases were assessed by nursing staff between 1 January and 31 December 2018.

### 2.7. Data Analysis

SPSS 22.0 was used for data analysis. Associations between demographic characteristics and fall incidence were assessed using Chi-squared tests, whereas a fall risk factor checklist and assessment scale were used for factor analysis. The assessment accuracy and cutoff point determination were evaluated using the receiver operator characteristic (ROC) curve, and the sensitivity, specificity, and positive and negative prediction rates for the fall risk factors were calculated to determine the odds ratio of fall risk prediction. The concordance of each assessment item between the old and new scales was tested to evaluate the equivalence of the improved scale. Finally, logistic regression analysis was used to determine the relationships between dependent variables and independent variables. The independent variables were the ten fall assessment items (see Table 3). The dependent variable was the absence or presence of a fall event.

### 2.8. Ethical Considerations

This study was approved by the institutional review board of the study hospital. Access to the medical records was authorized by the study hospital. The deidentification of medical records was performed prior to conducting the data analysis. To protect the rights and privacy of all patients, all data that met the inclusion criteria for data collection were stored safely and maintained confidential.

## 3. Results

The mean age of the sample population was 44.88 (SD = 12.05) years, and the mean duration of hospital stay was 44.04 (SD = 48.14) days. The study population was comprised of 66% men and 34% women.

### 3.1. Psychiatric Patient Background Variables

The background variables of the patients are shown in Table 1, which indicates that most of the patients were men, most were younger than 65 years, most had no experience with a prior fall occurrence, and most were classified as being at a high risk of falls.

### 3.2. Item Reduction, Reliability and Validity Analysis Results

Five items were removed from the original assessment according to the recommendations of expert reviewers. Items associated with fall detection factors were selected and subjected to an internal consistency and reliability analysis. The Cronbach’s alpha value of the scale was 0.74, indicating that the scale had good internal consistency after removing the identified items.

### 3.3. ROC Analysis Results

**Figure 2:**
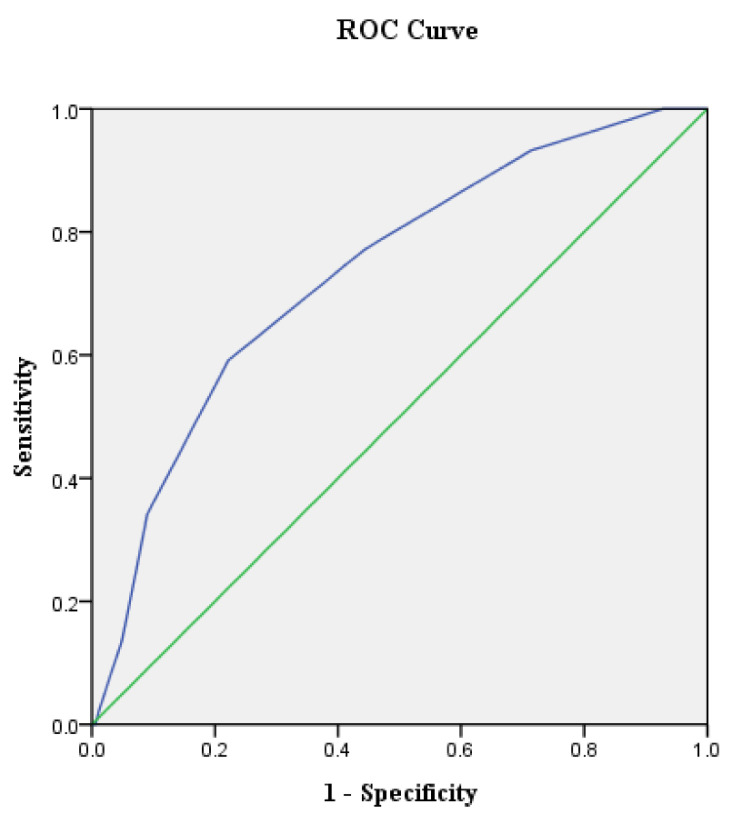
ROC curve analysis results of psychiatric patients.

The total score from each patient evaluation was used as the independent variable, and the falling occurrence was analyzed as the dependent variable. The results are shown in Table 2 and Figure 2. Using increased sensitivity and lower false positivity (1—Clarity) as a guideline, the optimal cutoff point for the scores associated with fall occurrence was determined to be 6.5 points. When the score is higher than 6.5 points, the patient is more likely to have experienced a fall occurrence. The area under the ROC curve (AUC) value for this study was 0.74 (*p* < 0.001), which is an acceptable discriminatory power according to the AUC standard, indicating the accuracy of using the score results to estimate fall occurrence (Table 2).

### 3.4. Logistic Regression Analysis Results

Most of the psychiatric patients consumed consciousness-altering drugs and psychiatric drugs, and none of the patients who experienced fall occurrences consumed diuretics or were leaving their beds for the first time following surgery or childbirth. Therefore, these four variables were removed from the assessment as being non-specific. The remaining assessment items were used as independent variables, and fall occurrence was used as the dependent variable. Logistic regression analysis was used to investigate the assessment factors that were more likely to affect fall occurrence. The model interpretation power was assessed, resulting in a Cox and Snell R^2^ value of 0.13 and a Nagelkerke R^2^ value of 0.46, with a prediction accuracy rate of 96.0% (Table 3). This study shows that the beta values of “consuming drugs that affect conscious activity”, “diuretics”, and “psychiatric medication” are 0 or tend toward infinity. Therefore, the variables cannot be presented, and these three items were not included in the logistic regression analysis.

The factors of “taking anti-epileptic drugs”, “the need for walking aids”, and “having experienced a fall occurrence within the previous year” were identified as significant predictors of fall occurrence. When a patient consumes anti-epileptic drugs, their chances of experiencing a fall occurrence was 2.98-times higher when they do not; when a patient requires walking aids, their chance of experiencing a fall occurrence was 2.58 times higher than when they do not; and when a patient has experienced a fall occurrence within the previous year, their chance of experiencing a fall occurrence was 178.61 times higher than when they do not.

The newly revised scale consists of a total of nine items. Among these items, “older than 65 years”, “unsteady gait or lower limb muscle strength ≤ 3 points”, and “consuming drugs that affect conscious activity” remained important factors that contributed to the likelihood of fall occurrence. Each of these items was worth 4 points, whereas the remaining items were worth 1 point each. The highest score was 18 points, and the lowest score was 0 points. The optimal cutoff point for fall occurrence was 6.5 points. After discussions with experts, patients scoring more than 6 points were classified as belonging to the high-risk, fall-prone group.

## 4. Discussion

This study successfully reduced the number of items used in the previous fall risk assessment scale from 14 to 9. The optimal cutoff point was reduced from 8 to 6 points, and the sensitivity and accuracy of the newly revised scale were satisfied. The revised scale accurately identified psychiatric patients that belong to the high-risk, fall-prone group.

In line with the global trend toward increased fall prevention among psychiatric patient groups, this current study used existing one-year data to modify and test a fall risk scale. The results of this study appear to be reliable and economically feasible for use in the regular assessment of potential fall risk among patients with psychiatric illnesses. This newly revised scale was used in the study hospital to detect early fall risks among in-hospital psychiatric patients. After accumulating additional data, such as five-year data, the psychometric properties may be re-examined using the larger dataset to enhance its accuracy and sensitivity for the application in hospital-based psychometric care.

Three factors were identified as significant predictors of fall risk among psychiatric patients in this study. First, the use of anti-epileptic drugs increases the chances of falling by three-fold compared to the non-use of such drugs. This finding differs from the results of a systematic review reported by Laberge and Crizzle [19], who identified a link between fall risk and the use of benzodiazepines and antidepressants, but no links were identified between fall risk and the use of antipsychotic drugs, antihypertensive drugs, anti-epileptic drugs, or alcohol. More evidence is required in the future to verify the important link between anti-epileptic drug use and fall occurrence. More importantly, psychiatric patients often suffer from ≥2 chronic diseases, and the percentage of psychiatric patients with more than one chronic physical condition is 52% greater than that for the general population [20]. When the number of chronic illnesses exceeds three, the rate of fall occurrence is even higher for this population [21]. Therefore, the hospital fall risk assessment scale should not only evaluate fall risk factors among psychiatric patients but also includes physical illness factors in the fall risk assessment scale, such as the use of anti-epileptic drugs and walking aids.

The chances of fall occurrence among patients who had experienced at least one fall within the previous year were 178-fold higher than the risk among patients who had not experienced a recent fall. By comparison, a study of the PIFRAT scale assessment found that the fall risk of those who had “experienced a fall in the past year” was 1.36 times higher than that of those who had not experienced a recent fall [17]. A similar finding was reported for another study that used the WSFRAT scale with patients who had “experienced a fall in the past year” being at a significantly higher risk of falling than those who had no experience of fall occurrence; among the patients in the first group, those with the highest risk of falling were patients “with a fall history within six months”, those who almost experienced a fall or were afraid of experiencing one, those who had experienced 1 or 2 falls, and those who had experienced more than two falls. The chances of fall occurrence among these various patient groups ranged from two to three times higher than those for other patient groups [17]. The scale used in this study should be able to effectively identify patients at an extremely high risk of falling, which will be helpful for the care of psychiatric patients. This study also recommends the use of an information management system to quickly assess whether a patient has any history of falling within the past year at the time of hospital admission, as well as the regular monitoring of fall occurrences among all patients.

This study found that the use of mobility aid equipment is related to fall occurrence. Previously, this variable has only been mentioned only in studies examining fall occurrence among older adults [22]. However, the use of walking aids among psychiatric patients was identified as a significant predictor of fall occurrence. Therefore, healthcare professionals and caregivers should be trained in the management of mobility aid equipment in the psychiatric ward, including the use of storage spaces for aid equipment and methods for correctly and safely assisting psychiatric patients with the use of such aids.

Some issues may be considered when applying the results from this study to nursing practice. This study was performed as a retrospective study that utilized patient medical records to test the psychometric properties of a psychiatric fall risk assessment instrument. Although the methodology may be similar to Edmonson, Robinson and Hughes [14], the dataset used in this study was larger than that used for the EPFRAT. Furthermore, after accumulating additional data, such as five-year data, these psychometric properties may be re-examined using the larger dataset to enhance the accuracy and sensitivity of this scale for applications in hospital-based psychometric care. Furthermore, the findings from this study may have been influenced by cultural factors, as is the case with the link between the use of mobility aid equipment and increased fall occurrence. Future research remains necessary to examine particular aspects of the issue of falls among psychiatric patients.

Limitations. The methodology and data collection strategy applied in this study may represent potential limitations. First, the number of patients who experienced falls was much smaller than the number of patients who did not experience any falls. Therefore, the findings of this study might be overstated, especially with respect to the predictions of fall probability. Second, some key information could not be analyzed because the original data were not clearly reported. Additionally, this study collected cases from only one psychiatric hospital in Taiwan, and the generalizability of the findings from this study may, thus, be limited. We view these results as preliminary, requiring further confirmation before they can be applied on a wider scale. In addition, the findings from this study may have been influenced by cultural factors, as is the case for the association between the use of mobility aid equipment and increased fall occurrences.

## 5. Implications for Practice

Several recommendations are proposed based on these study results. (a) The rate of hospitalization for recurring psychiatric conditions is high. Therefore, as soon as a patient is admitted into the hospital, the hospital’s information screen should provide an alert regarding whether they have any history of fall occurrence within the past year, which can rapidly identify those at a high risk of fall occurrence and provide staff with preventive measures for avoiding future fall occurrence. (b) The psychiatric nursing unit must formulate a comprehensive mobility aid equipment management plan, encompassing mobility aid equipment rental, adequate storage space, lines of movement, maintenance and repair procedures, timely inspection, and the elimination of outdated aid equipment systems. (c) Psychiatric wards should strengthen the caregiving skills of nursing staff and support staff in assisting users with aid equipment. (d) The education and training of newly incoming nurses, the reinforcement of fall assessment skills, and fall prevention strategies should be conducted by nursing staff in stages to integrate empirical evidence into fall prevention training, which can help psychiatric nurses maintain up-to-date knowledge and allow them to apply effective strategies for fall prevention. (e) A liaison consultation may identify drugs for patient use that may be safer in terms of the effects on fall risk.

## 6. Conclusions

This study aimed to test the psychometric properties and modification of an existing fall risk assessment scale and examine the fall predictors associated with psychiatric patients. A concise and effective fall risk assessment scale was successfully established. Three predictors for fall occurrence among psychiatric patients were identified. To avoid repeated falls, the hospital information system should provide alerts regarding each patient’s fall history, and information regarding the use of anti-epileptic drugs and mobility aids should be included in fall risk assessment scales. On-the-job training among nursing staff should integrate empirical evidence regarding practical applications for fall prevention and other similar contents, which can help nurses to accurately and rapidly identify psychiatric patients at high risk of falling.

## Figures and Tables

**Table 1:** Psychiatric patient background variables (N = 1101).

Variable	Category	Frequency	Percentage (%)
Gender	Female	374	34.0
	Male	727	66.0
Age > 65	No	1050	95.4
	Yes	51	4.6
Experience of fall occurrence	No	1057	96.0
	Yes	44	4.0
Frequency of fall occurrence	Evaluation within 24 h	21	1.9
	Evaluation within 24 h and 3 days	75	6.8
	Evaluation within 24 h, 3 days, and 7 days	1005	91.3
High-risk group	No	422	38.3
	Yes	679	61.7

**Table 2:** ROC analysis of psychiatric patients.

Score Results	Sensitivity	1–Clarity (Specificity)
0.0000	1.000	1.000
1.5000	1.000	0.999
2.5000	1.000	0.995
3.5000	1.000	0.928
4.5000	0.932	0.712
5.5000	0.773	0.445
6.5000	0.591	0.221
7.5000	0.341	0.090
8.5000	0.136	0.049
9.5000	0.023	0.011
10.5000	0.000	0.006
11.5000	0.000	0.001
13.0000	0.000	0.000

**Table 3:** Logit model of psychiatric patients.

No.	Assessment Items	B	S.E.	Wals	Significance	Exp(B)	95% CI	
							lower bound	lower bound
1.	Older than 65 years (No = 0)	1.29	0.76	2.89	0.09	3.65	0.820	16.209
2.	Unsteady gait or 3 points for lower limb muscle strength (No = 0)	0.37	0.84	0.20	0.66	1.45	0.278	7.559
3.2	Laxatives (No = 0)	−0.42	0.44	0.92	0.34	0.66	0.276	1.555
3.3	Sedatives (No = 0)	−0.43	0.39	1.19	0.28	0.65	0.304	1.403
3.4	Lowered blood pressure (No = 0)	−0.69	0.47	2.14	0.14	0.50	0.201	1.262
3.5	Lowered blood sugar (No = 0)	−0.017	0.49	0.13	0.72	0.84	0.325	2.180
3.6	Anti-epileptic drugs (No = 0)	1.09	0.43	6.43	0.01 *	2.98	1.282	6.943
4.	Consciousness disturbance: 714 points on the GCS (No = 0)	−0.20	1.32	0.02	0.88	0.82	0.061	10.883
5.	Physical weakness, dizziness, and migraine (No = 0)	0.54	0.50	1.16	0.28	1.72	0.641	4.627
6.	Need for walking aids (No = 0)	0.95	0.47	4.03	0.05 *	2.58	1.022	6.504
7.	Need to leave the bed frequently to use the restroom, ≥8 times in the daytime, and ≥2 times at night (No = 0)	−0.65	0.93	0.49	0.49	0.52	0.084	3.242
8.	No caregivers during hospitalization (No = 0)	1.06	1.15	0.86	0.35	2.89	0.307	27.275
**9.**	Has experienced fall occurrence within one year (No = 0)	5.19	1.02	25.65	<0.01 ***	178.61	24.013	1328.597
**10.**	High-risk (No = 0)	−0.97	0.90	1.16	0.28	0.38	0.066	2.202
	Cox & Snell R^2^			0.13				
	Nagelkerke R^2^			0.46				
	Accuracy of prediction			96.0%				

* *p* < 0.05, *** *p* < 0.001; the category “No” is the reference group.

## Data Availability

Data sharing not applicable to this article as no datasets were generated or analyzed during the current study.
